# Cardiac Tamponade and Myocardial Infarction: A Case Report

**DOI:** 10.7759/cureus.63284

**Published:** 2024-06-27

**Authors:** S Angel, Venkatesan M, Priyadarshee Pradhan

**Affiliations:** 1 Forensic Medicine and Toxicology, Sri Ramachandra Institute of Higher Education and Research, Chennai, IND

**Keywords:** hypertension, rupture of ventricles, cardiac tamponade, acute myocardial infarction, sudden death

## Abstract

In recent times, there has been a concerning rise in the incidence of sudden death among individuals in middle age. Predominantly, cardiovascular diseases emerge as the leading cause behind such untimely fatalities. Myocardial infarction and its subsequent complications stand out as the most frequently encountered scenarios in these tragic events. Despite being a relatively uncommon occurrence, cardiac tamponade represents one of the rare yet fatal complications that can ensue following a myocardial infarction. This condition manifests when the pericardial cavity becomes filled with either blood or blood clots, impeding the heart’s normal functioning. Typically, patients experiencing cardiac tamponade are often reported to have succumbed to sudden death, with a preceding history of chest pain being a common indicator. The definitive diagnosis of cardiac tamponade usually occurs during post-mortem examinations. We consider the case of a 38-year-old man who was discovered unconscious at his residence and was pronounced dead upon arrival at the hospital. Subsequent autopsy findings unveiled the presence of both blood and blood clots within the pericardial cavity, in conjunction with a rupture in the right ventricle and occlusion of the left coronary artery. Histopathological analysis further confirmed the root cause of this tragic event as an acute myocardial infarction.

## Introduction

Cardiac tamponade is a life-threatening medical condition often reported to the ED as sudden death. This occurs due to the accumulation of blood within the pericardium, which impedes heart function. The heart is covered by the pericardium, and the pericardial cavity contains about 30-50 ml of thin, straw-colored fluid that provides cushioning and acts as a protective covering. Apart from this straw-colored fluid, an accumulation of additional fluid is termed pericardial effusion; blood is called hemopericardium, and pus is known as purulent pericarditis [1].

If the volume of blood exceeds 150 ml, there will be an increase in intrapericardial pressure that exceeds the central venous pressure. This increase in pressure can be either gradual or sudden, depending on the rate of blood accumulation, leading to compromised venous return to the heart and a subsequent decrease in cardiac output. It also affects coronary circulation. The major causes of cardiac tamponade include traumatic events like road traffic accidents and stab injuries to the chest, which lead to laceration of the myocardium or aorta, resulting in acute filling of the cavity with blood. Non-traumatic causes include pericarditis, iatrogenic factors such as invasive procedures on the heart, collagen diseases like systemic lupus erythematosus, and post-myocardial infarction [2-4].

It is often a fatal condition that presents with chest pain, dyspnoea, and tachypnoea, and ultimately, collapse due to cardiogenic shock. Immediate intervention is required. The blood can fill the pericardial cavity acutely or gradually. Acute filling of 200-300 ml of blood will be fatal, leading to death in traumatic cases because there is no time for immediate intervention, thus preventing any compensatory mechanism from averting circulatory collapse. A gradual filling allows time to treat the patient, who will present with the aforementioned signs and symptoms. Radiologically, it can be identified by an X-ray, which shows a globular shadow with normal lungs. Other investigations include echocardiography, fluoroscopic scanning, and radioisotope scanning. Most of the time, the patient will present as sudden death to the emergency department, as cardiovascular causes are known to be the leading cause of sudden death. Cardiac tamponade accounts for 20%-30% of these cases. Rapid onset cases are often diagnosed during autopsy [5-8].

## Case presentation

A 38-year-old male was taken to a private hospital casualty in an unconscious state by his wife at around 4:50 AM on April 19, 2024, after he had been unresponsive for over 15 minutes at his residence. At the casualty, he was examined and declared dead at 5:15 AM. He had a history of abdominal pain and vomiting, for which he had received treatment at a nearby clinic, and he was also advised to take an ECG, which he did not undergo. He had a history of a transient ischemic attack (TIA) that led to right-sided hemiplegia two years earlier. He was treated for that and was on hypertensive drugs, which he had discontinued on his own six months prior. The case was brought for autopsy.

**Figure 1:**
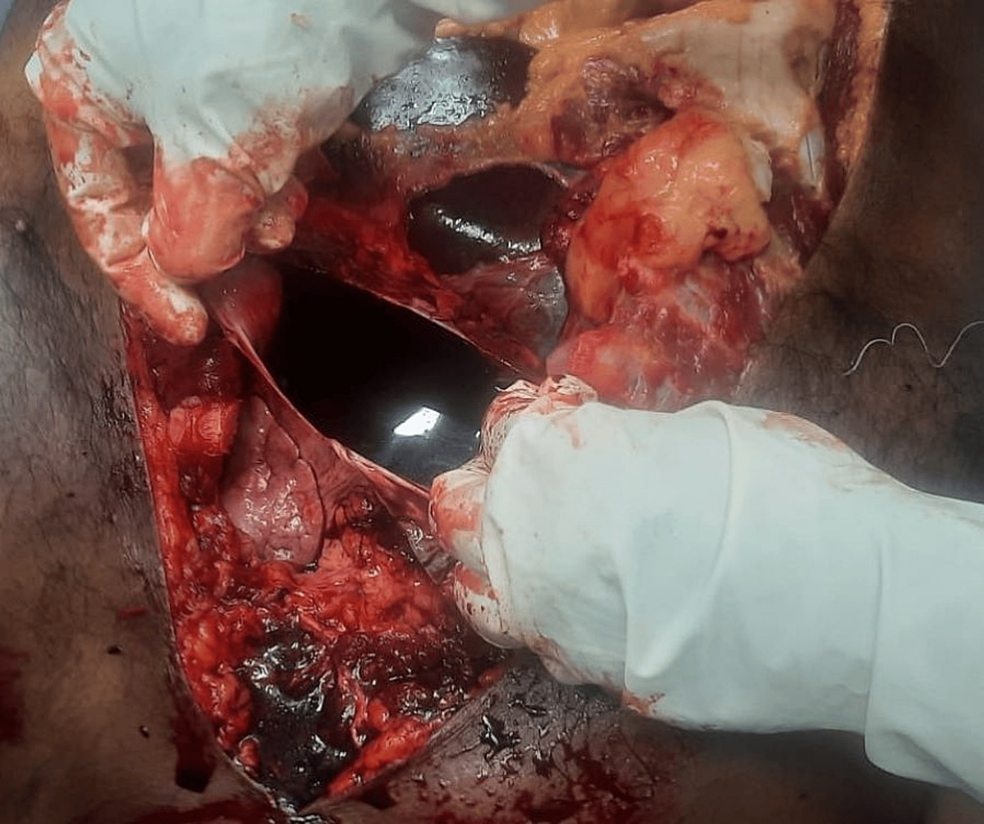
Pericardial cavity filled with blood-stained fluid. The figure shows the pericardial cavity filled with 250 ml of blood-stained fluid, which is visible immediately after dissecting the pericardium.

**Figure 2:**
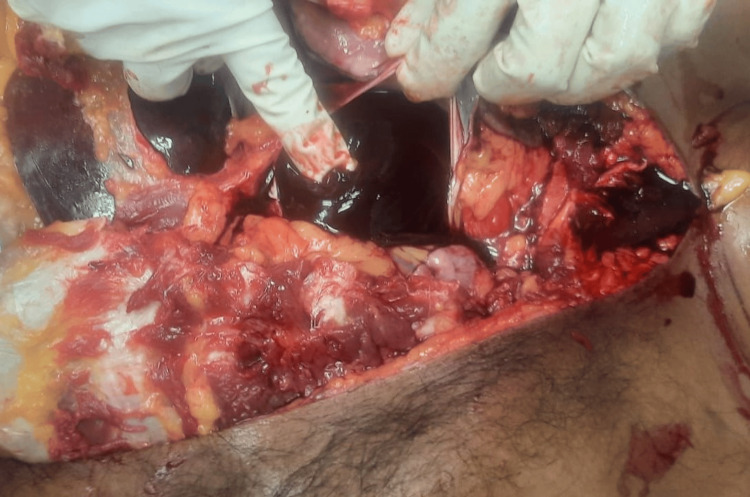
Pericardial cavity with blood clot. The figure shows the pericardial cavity containing an 180-gram blood clot, weighed using a weighing machine.

**Figure 3:**
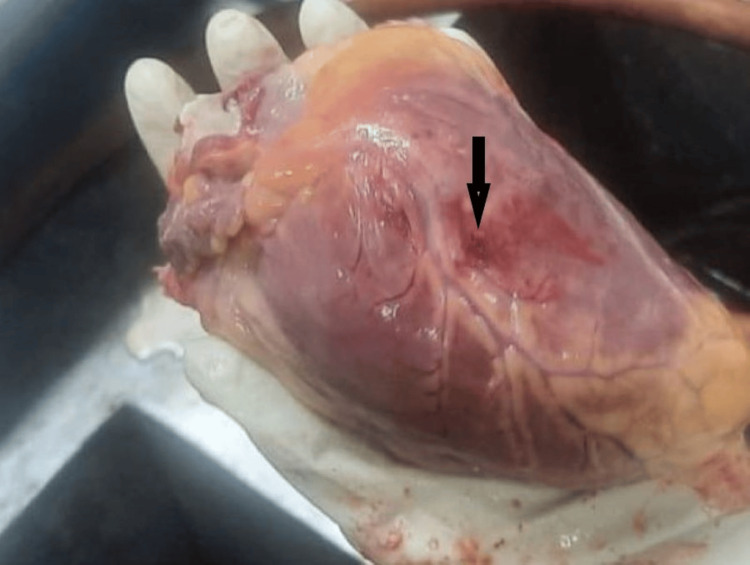
Myocardium with rupture. The figure shows a myocardial rupture, indicated by an arrow.

The autopsy was conducted on the same day at 2:30 PM. On external examination, both conjunctivae were congested, and postmortem hypostasis was fixed. On internal examination, as soon as we started to dissect the pericardium, we observed that the pericardial cavity was filled with blood. It contained about 180 grams of blood clot and 250 ml of blood-stained fluid (Figures 1-2). After dissecting the pericardium, the heart was removed and weighed 410 grams. It was found that there was a rupture measuring 0.5 cm x 0.5 cm on the posterior aspect of the left ventricle (Figure 3). The heart was dissected using the inflow and outflow method, which showed that the coronaries were occluded to pinhead size, atheromatous plaques were present over the root of the aorta, and all the valves were calcified. The left ventricle wall thickness was 2.5 cm. All other organs were dissected and showed congestion.

**Figure 4:**
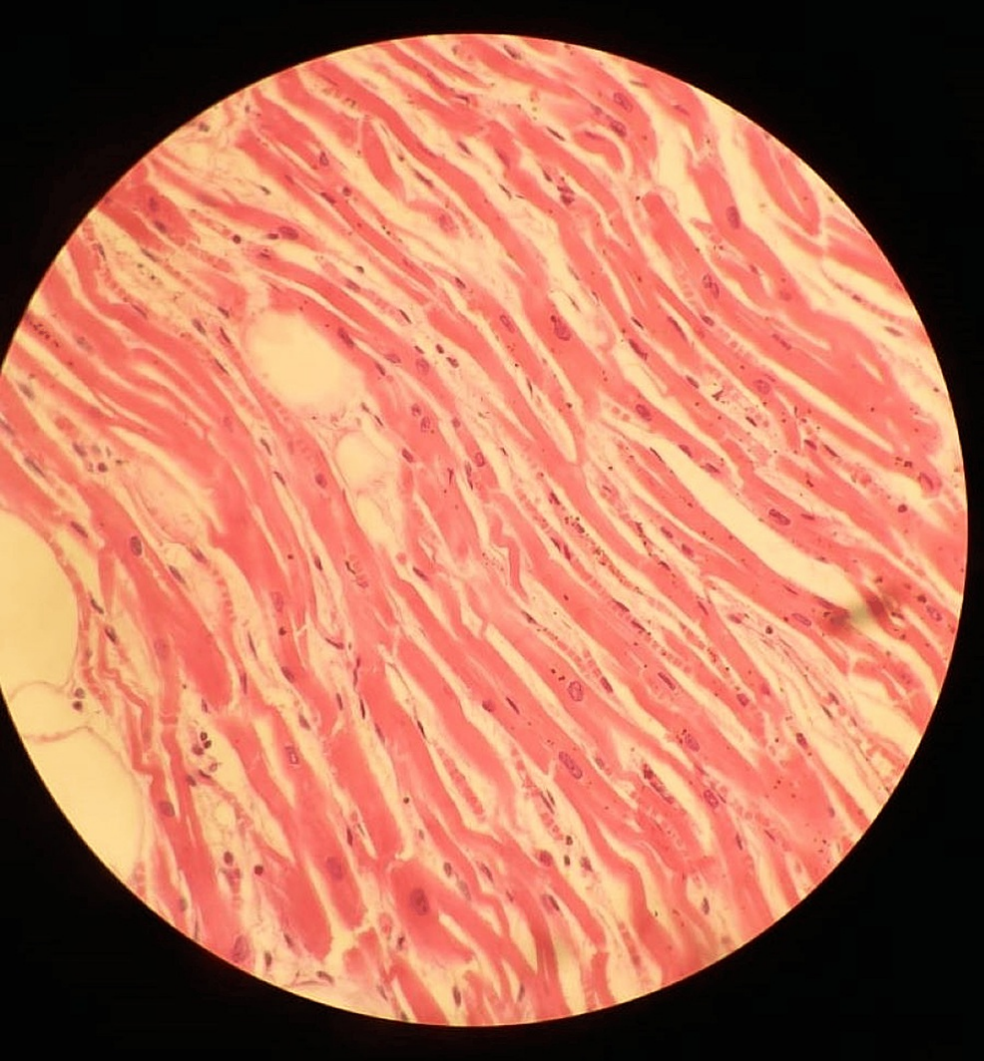
Histopathology showing early myocardial infarction. The figure shows the histopathology image showing the waviness of myocardial fibres with neutrophilic infiltrate, giving the final impression of acute myocardial infarction.

Sections of myocardium from the rupture site and the left anterior descending artery were preserved in formalin. Histopathological examination of the myocardium showed neutrophilic infiltrate with waviness of fibers and focal necrosis. These features suggest acute myocardial infarction (Figure 4). The coronaries showed luminal narrowing with the formation of atheromatous plaque and recanalization. Thus, the opinion has been given that the deceased died due to cardiac tamponade.

## Discussion

Cardiac tamponade, a condition characterized by the accumulation of fluid in the pericardial sac, represents a significant contributor to instances of sudden death, frequently identified during post-mortem examinations in both traumatic and non-traumatic scenarios. While relatively rare, it stands out as one of the most fatal complications associated with acute myocardial infarction. The dangers posed by cardiac tamponade lie in its potential to precipitate the rupture of vital cardiac structures such as ventricles, septum, and papillary muscles. Studies suggest that as much as 15% of premature fatalities linked to myocardial infarction can be attributed to the insidious onset of cardiac tamponade. The insidious nature of this condition underscores the critical need for timely diagnosis and intervention to avert its catastrophic consequences [7-9].

In the present case, the deceased individual was identified as having a history of hypertension and was irregularly following a prescribed treatment regimen. Further examination revealed the presence of an occlusion in the left coronary artery and a rupture of the myocardium. These findings indicate that the cardiac tamponade experienced by the individual resulted from complications arising from a myocardial infarction, a conclusion that has been substantiated by histopathological analysis. It is essential to note that this occurrence represents a rare and grave complication with severe consequences. Similar findings have been noted in many cases, along with a history of irregular medication for hypertension and diabetes, which further complicates the myocardial infarction.

Sonwani NS et al. presented findings from a study detailing three cases of cardiac tamponade, a serious condition in which the heart is compressed by fluid accumulation in the pericardial sac. In all three cases, the patients exhibited evidence of either thrombus formation or complete occlusion in the coronary arteries, leading to the rupture of the ventricles. This rupture of the heart chambers due to increased pressure from the tamponade can result in life-threatening consequences if not promptly addressed [3]. Patil A et al. documented a case involving the sudden death of a 50-year-old man with a medical history of diabetes mellitus and hypertension, who was not consistently following his prescribed treatment regimen. An autopsy revealed that the cause of death was cardiac tamponade, attributed to the occlusion of the left circumflex coronary artery [4]. Thakral S et al. discussed two instances of individuals aged 60 years who succumbed to cardiac tamponade. Both cases exhibited significant atherosclerotic changes in the coronary arteries, underscoring the potential role of underlying cardiovascular conditions in predisposing individuals to this life-threatening emergency [5].

The observations from the above-mentioned cases highlight a common trend where individuals who have passed away due to cardiac tamponade often had a history of hypertension or diabetes but were not undergoing consistent treatment. In the specific instance of the deceased individual in this study, it is notable that he was of middle age. This aspect serves as a crucial revelation, indicating that cardiac tamponade is not exclusive to the elderly population but can also manifest in middle-aged individuals who are not receiving appropriate medical care. An individual with pre-existing hypertension may experience an increased sympathetic response to the accumulation of blood, resulting in a further surge in blood pressure [6]. Such a physiological response exacerbates the patient's condition, eventually leading to a state of shock. It is imperative to acknowledge that cardiac tamponade, while a rare complication, is potentially preventable through the consistent management of hypertension or diabetes with prescribed medications and also with consistent follow-up [10-13].

Cardiac tamponade, while rare, represents one of the most fatal complications that can arise from a myocardial infarction. As such, it becomes imperative to ensure that the autopsy process following any sudden death case is conducted with the utmost care and meticulous attention to every detail to ascertain the presence of a potential rupture. This thorough examination becomes even more crucial as it allows for a histopathological analysis to be performed, unveiling the underlying pathology responsible for the development of cardiac tamponade. Through a comprehensive investigation that includes both macroscopic observations and microscopic analysis, medical professionals can gain a deeper understanding of the mechanisms occurring within the heart, aiding in the detection and management of such life-threatening conditions [14-17].

## Conclusions

In the case being discussed, the individual who passed away was middle-aged, and the cause of death was attributed to cardiac tamponade. Upon examination, evidence of ventricular rupture was observed, and subsequent histopathological analysis confirmed the presence of acute myocardial infarction. The findings indicate that unmanaged hypertension probably had a substantial impact on the blockage of coronary arteries, thereby exacerbating the person's health issues. It is crucial to carry out a detailed postmortem examination along with a comprehensive analysis of the deceased individual's medical documentation to reveal the fundamental pathological factors that led to this result. By thoroughly investigating the individual's medical history and conducting a thorough autopsy, medical professionals can gain insights into the precise mechanisms through which uncontrolled hypertension contributed to the occlusion of coronary vessels, ultimately aiding in the understanding and prevention of similar complications in the future.
